# Crystal Structures of the Catalytic Domain of Human Soluble Guanylate Cyclase

**DOI:** 10.1371/journal.pone.0057644

**Published:** 2013-03-07

**Authors:** Charles K. Allerston, Frank von Delft, Opher Gileadi

**Affiliations:** Structural Genomics Consortium, University of Oxford, Oxford, The United Kingdom; Institute of Enzymology of the Hungarian Academy of Science, Hungary

## Abstract

Soluble guanylate cyclase (sGC) catalyses the synthesis of cyclic GMP in response to nitric oxide. The enzyme is a heterodimer of homologous α and β subunits, each of which is composed of multiple domains. We present here crystal structures of a heterodimer of the catalytic domains of the α and β subunits, as well as an inactive homodimer of β subunits. This first structure of a metazoan, heteromeric cyclase provides several observations. First, the structures resemble known structures of adenylate cyclases and other guanylate cyclases in overall fold and in the arrangement of conserved active-site residues, which are contributed by both subunits at the interface. Second, the subunit interaction surface is promiscuous, allowing both homodimeric and heteromeric association; the preference of the full-length enzyme for heterodimer formation must derive from the combined contribution of other interaction interfaces. Third, the heterodimeric structure is in an inactive conformation, but can be superposed onto an active conformation of adenylate cyclase by a structural transition involving a 26° rigid-body rotation of the α subunit. In the modelled active conformation, most active site residues in the subunit interface are precisely aligned with those of adenylate cyclase. Finally, the modelled active conformation also reveals a cavity related to the active site by pseudo-symmetry. The pseudosymmetric site lacks key active site residues, but may bind allosteric regulators in a manner analogous to the binding of forskolin to adenylate cyclase. This indicates the possibility of developing a new class of small-molecule modulators of guanylate cyclase activity targeting the catalytic domain.

## Introduction

Soluble guanylate cyclases (sGC) (EC 4.6.1.2) are nitric oxide (NO) sensors which, when activated, catalyse the conversion of GTP to the second messenger cGMP. The downstream signals propagated through cGMP are varied and include peristalsis of the gut, vascular smooth muscle tone and blood pressure, aortic relaxation, brain development and platelet aggregation [1]. cGMP is also produced by particulate guanylate cyclases, which are transmembrane receptors responding to hormones other than NO.

In mammals, four isoforms of sGC subunits have been identified so far: α1, α2, ß1 and ß2. *GUCY1B3* encodes the ß1 subunit of sGC which can form heterodimers with either of the α subunits [2]. *GUCY1A3* encodes the α1 subunit (sGCα and sGCβ denote the protein subunits of soluble guanylate cyclase encoded by the *GUCY1A3* and *GUCY1B3* genes; sGCαcat and sGCβcat denote the isolated catalytic domains. AC-C1 and AC-C2 are the two catalytic domains of mammalian adenylate cyclase, which are the equivalent of sGCαcat and sGCβcat, respectively). The predominant complex is the α1/ß1 dimer, which is ubiquitously expressed, with highest levels of mRNA in brain, lung, heart, kidney, spleen and muscle [3], [4]. The phenotype of *GUCY1B3* knockout mice confirms the central role of this gene in many physiological processes. Mice die 3–4 weeks after birth with pronounced elevation in arterial blood pressure and impaired NO-induced relaxation. Mice have severe gastrointestinal dysfunction with intestinal dysmotility. In addition knockout mice showed reduced nociceptive behavior in models of inflammatory or neuropathic pain [5], [6]. Overexpression of *GUCY1A3* and *GUCY1B3* has also been reported in some glioma cell lines and is linked to neo-vascularisation in these tumors [7]. It is important to note, that although the active form of sGC in mammals is a heterodimer, in lower organisms (e.g. nematodes, algae, and insects) active homodimers, active heterodimers and even one case of an inactive heterodimer have been reported [8], [9], [10], [11].

**Figure 1:**
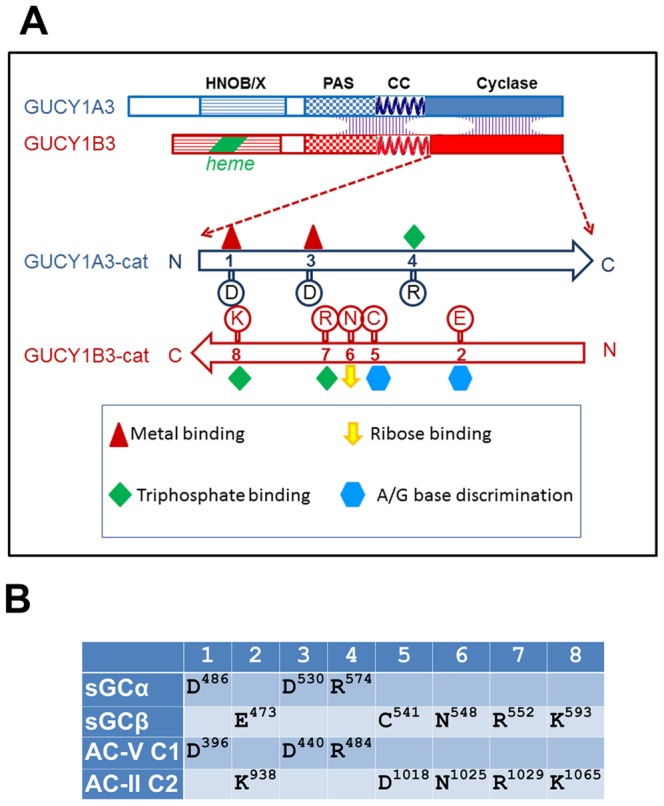
Structural organization of guanylate cyclase. A. Schematic depiction of domain organization of human guanylate cyclase. The native enzyme is a heterodimer of α and β subunits (encoded by GUCY1A3 and GUCY1B3, respectively), with interaction interfaces spread across the PAS, CC and cyclase catalytic domains. The catalytic domains associate in a head to tail orientation; conserved residues involved in substrate binding and catalysis are distributed between the two subunits. B. A list of conserved active-site residues in guanylate cyclase (sGCα and β) and in the chimeric adenylate cyclase (AC-V C1 domain and AC-II C2 domain). A full alignment of adenylate and guanylate cyclases is shown in Supporting Fig. S1.

Each sGC subunit is a multi-domain protein (Fig. 1A). An N-terminal haem-binding domain in sGCβ is termed H-NOX. The corresponding domain in the α subunit cannot bind haem and is denoted H-NOB. The H-NOX serves as a sensor of NO which binds to the prosthetic haem group, inducing a conformational change which propagates the signal and results in greatly increased cGMP production by sGC [12], [13]. PAS-like domains in both subunits are thought to influence the preference of mammalian sGC for heterodimer formation [14]. Further downstream, a predicted coiled-coil bundle is followed by the C-terminal catalytic domains, where the GTP binding and conversion takes place.

sGC belongs to the class III purine nucleotidyl cyclase family, along with adenylate cyclases (AC), which perform an analogous function of converting ATP to the second messenger cAMP. Both AC and GC catalytic domains share a basic core of secondary structure elements that dimerise to form a wreath-like structure. The active sites of both adenylate and guanylate cyclases lie at the interface between the two homologous subunits or subdomains, and comprise residues from both subunits. In the homodimeric sGC of lower organisms, there could be in principle two symmetry-related active sites. However, in the active conformation, only one site is arranged to accommodate a substrate. In heteromeric cyclases such as human sGC and AC, the active site contains all the residues required for substrate recognition and catalysis. The pseudosymmetry-related cavity, which lacks key catalytic residues, has been suggested as a regulatory site [15], [16], [17] and is the site of action of the AC activator forskolin [18], [19], [20].

Guanylate cyclase is a major drug target as it is the main receptor for vasodilating NO-releasing compounds, which have been used for over 100 years in the treatment of coronary heart disease and angina pectoris. There are two drugs currently in late drug development, Riociguat and Cinaciguat, both developed by Bayer. They are classified as sGC stimulators and activators, respectively, working by binding at the haem binding domain of the ß subunit of sGC [21], [22], [23]. In addition to these two drugs and the large number of small molecule progenitors which led up to them over the course of several decades, only two chemically related sGC inhibitors are currently under investigation, namely ODQ [24] and NS-2028 [25], [26]. These molecules are also thought to act by oxidation and desensitisation of the heam group to NO. A growing body of evidence has implicated sGC as a player in the angiogenesis pathway [7], [25], [27], [28], [29], [30], a popular pathway to drug in the inhibition of tumor vascularisation [31], [32] and to combat diabetic retinopathy [33], [34]. An as-yet unexploited avenue for pharmaceutical development would be targeting the catalytic domain for activation or inhibition. With this consideration, the atomic structure of sGC catalytic domain (sGCcat) molecule would therefore be extremely desirable with a view towards rational drug design.

Despite a great deal of research on mammalian sGC over the past three decades, all structural analysis is derived from modeling based on structures of other nucleotidyl cyclases. Published structures of guanylate cyclases include homodimeric enzymes of the single-celled green alga *Chlamydomonas reinhardtii* [10] with a primary sequence identity of 39% and 41% to the human α and ß sGCcat, respectively; and the homodimeric sGCcat (which has concurrent AC activity) from the cyanobacterium *Synechocystis PCC6803* [35], which is 22% identical to the human alpha and beta sGCcat. The only heterodimeric structures are of adenylate cyclases, all of which are a non-natural hybrid of domains from the AC2 and AC5 proteins (Protein Data Bank ID: 1CS4 [36], 3C14 [37], 2GVZ [38], 1AZS [18], 1CJU [39].

We present here two structures of human sGC catalytic domains. The first is a homodimeric form of sGCßcat. The second is a heterodimeric form of sGCαcat and sGCβcat. We discuss the catalytic properties of sGCcat and compare structures of ACcat and sGCcat to indicate probable mechanisms and domain motion during catalysis. Finally we discuss these structures in terms of further research and drug development opportunities.

## Materials and Methods

### DNA and protein engineering

DNAs encoding human guanylate cyclase were designed and synthesized with a codon distribution optimized for expression in E. coli (Codon Devices). The catalytic domain of GUCY1A3 (aa 468–690) was inserted into vector pNH-TrxT (GenBank:GU269914) using ligation-independent cloning [40]; this generates a fusion protein with an N-terminal his_6_-tagged thioredoxin gene followed by a TEV protease cleavage site. The catalytic domain of GUCY1B3 (aa 408–619) was cloned into vector pNIC-CTHF (GenBank: EF199844), which generates a fusion protein with a C-terminal TEV cleavage site followed by a his_6_ and Flag tag.

A construct of GUCY1B3 with the amino acid substitutions G476C, C541S was generated by site-directed mutagenesis. The nucleotide sequences of the expression constructs of GUCY1A3, GUCY1B3, and the GUCY1B3- G476C/C541S mutant are deposited with GenBank accession numbers JX420281, JX420283, and JX420282, respectively.

### Protein purification

Protein expression and purification were performed as previously described [40], with the following modifications. To assist recovery of soluble protein, the expression vector was transformed into BL21(DE3)-R3 cells containing plasmid pGro7 (TaKaRa), which provides arabinose-inducible expression of the bacterial chaperone genes GroEL and GroES. Bacterial host cells bearing either *GUCY1A3* or *GUCY1B3* were grown in Terrific Broth at 37°C to OD^600^ of 1; expression of the chaperone proteins was then induced with 0.2% L-arabinose for 1 hour. The cultures were then transferred to 18°C, and after one hour IPTG was added to 0.5 mM. Growth was continued overnight.

sGCß was purified as described [40] by Ni-affinity purification followed by gel filtration on a Superdex 75 column, which resulted in separation of most of the dimeric sGC protein from the co-expressed chaperones and other host proteins. Further purification was achieved by cleaving the tag with TEV protease; the protease and other impurities were removed by passing through Ni-IDA column (Generon). The protein was then concentrated to 12 mg/ml.

For purification of heterodimers of sGCα and mutant sGCβ ^G476C/C541S^, the two proteins were expressed separately as described above. The cell pellets were combined and lysed together by sonication; all buffers were prepared without reducing agents. Following affinity purification on NiNTA, the eluted proteins were combined with TEV protease in a dialysis tubing (30 kDa cutoff) and dialyzed overnight against affinity buffer [40]. The protease and other impurities were removed by passage through a Ni-IDA column; the sGC protein was then purified by gel filtration (Superdex 75) equilibrated in 50 mM HEPES, pH 7.5, 300 mM NaCl, 5% glycerol. The heterodimers were diluted 15-fold with 25 mM tris-HCl, pH 8.5 and purified by ion exchange chromatography on a 5-ml Q XL column (GE Healthcare). The column was equilibrated in buffer A (25 mM tris-HCl, pH 8.5, 20 mM NaCl), the protein was loaded at 4 ml/min, and eluted with a 200-ml gradient to buffer B (25 mM tris-HCl, pH 8.5, 1.0 M NaCl). A main peak eluting around 270 mM NaCl contained both subunits; a minor peak, containing only sGCα, eluted at lower salt.

Fractions containing the heterodimer were pooled, exchanged to 25 mM tris-HCl, pH 8.5, 150 mM NaCl, and concentrated to 16 mg/ml.

### GC activity assay

Guanylate cyclase activity was assayed in a buffer containing 50 mM tris-HCl, pH 7.5, 3 mM MgCl_2_ or MnCl_2_ as indicated, 0.5 mg/ml BSA, 0.5 mM unlabeled GTP, 0.1 µCi/µl of [α-^32^P]-GTP, and 1–1000 µM protein. Where indicated, spermine-NO (Sigma S150) was added to 2.5 µM from a 100 µM solution in 10 mM NaOH. 10-µl reactions were incubated at 37°C for 15 minutes. Aliquots (1 µl) were spotted on PEI-cellulose thin layer chromatography sheets (Merck) and developed with a solution of 50 mM MES-NaOH, pH 7.0, 0.66 M LiCl. Spots corresponding to GTP and cGMP were quantitated using a Kodak phosphorimager screen K and a BioRad PMI analyzer. All experiments were performed in triplicate. Full-length soluble guanylate cyclase (bovine lung) from Enzo Life Sciences was kindly provided by John Garthwaite (University College, London).

### Crystallization

Crystals of the homodimeric sGCβ were grown by vapour diffusion at 4°C in 150 nl sitting drops. The drops were prepared by mixing 100 nl of protein solution and 50 nl of precipitant consisting of 0.1 M HEPES, pH 7.5, 1.4 M Na_3_ citrate. Crystals of the heterodimeric sGCα/sGCβ were grown by vapour diffusion at 20°C in 150 nl sitting drops. The drops were prepared by mixing 75 nl of protein solution and 75 nl of precipitant consisting of 0.05 M KH_2_PO_4_ and 20% PEG 8000. The crystals were cryo-protected using the well solution supplemented with 25% ethylene glycol and flash-frozen in liquid nitrogen.

### Data Collection and structure solution

Diffraction data for the homodimeric form of sGCβ and the heterodimeric complex formed between the catalytic domain of sGCα and the mutated (G476C/C541S) catalytic domain of sGCβ were collected from a single crystal. Diffraction intensities were integrated using Mosflm [41] and scaled and merged using SCALA [42]. The structure of the sGCβ homodimer was phased using the molecular replacement module of Phaser with the catalytic domain of the sGCcat of *C.reinhardtii* [10] (Protein Data Bank code (PDB) ID: 3ET6) as the search model. Model building and refinement was performed using COOT [43], REFMAC [44] and Phenix.refine [45]. TLS groups were selected using the TLSMD server [46]. The deposited structure was assigned the PDB code 2WZ1 (table 1).

**Table 1:** Crystallographic statistics.

Data Collection	ß Homodimer 2WZ1	α1/ß1 Heterodimer 3UVJ
**Beamline**	Diamond I03	Diamond I04
**Wavelength (Å)**	0.9795	0.9686
**Space Group**	C 2 2 21	P 21
**Unit Cell**	a = 65.59, b = 90.38 , c = 140.03 α = 90, β = 90, γ = 120	a = 50.78, b = 139.1, c = 55.46 α = 90, β = 91.47, γ = 90
**Resolution (Å)**	37-1.63 (1.67-1.63)	51.5-2.08 (2.16-2.08)
**Rmerge (%)**	6.5 (56.8)	9.1 (60.6)
**I/σ (I)**	9.9 (2.1)	8.6 (2.0)
**Completeness (%)**	99.3 (99.8)	99.5 (99.7)
**Redundancy**	3.4 (3.5)	3.3 (3.3)
**Refinement**		
**Unique reflections**	49220	45891
**Free R test set (%)**	5	5
**Rwork/Rfree**	0.219/0.185	0.209/0.169
**Dimers per A.U.**	1	2
**No. atoms**	3431	6267
**Protein**	3130	5804
**Ligand**	12	32
**Solvent**	289	431
**r.m.s. deviation, bond lengths (Å)**	0.016	0.010
**r.m.s. deviation, bond angles (Å)**	1.585	1.02

The structure of the heterodimeric complex was phased and initial models were built using the BALBES web server [47]. Iterative rounds of manual rebuilding and refinement were performed using COOT and BUSTER [48]. The deposited structure was assigned the PDB code 3UVJ (table 1). Stereochemical validation of the structures was performed using the Molprobity server [49].

To model the ligand-bound, closed conformation of sGCcat, the heterodimer was superimposed over the ACcat heterodimer containing the ATP analogue 2′,3′-dideoxyadenosine triphosphate (ddATP) [18]. The ß subunit of sGCcat and C2 domain of ACcat were used superimpose the structures, then the α subunit of sGCcat was independently superimposed on the closed C1 domain of sACcat. Analysis of protein domain movement was performed using DynDom [50].

## Results

### Structure overview

The full-length guanylate cyclase (sGC) protein forms a heterodimer of the homologous α1 and β1 subunits, encoded by the *GUCY1A3* and *GUCY1B3* genes, respectively. The domain organization is depicted schematically in fig. 1A. Mutational and structural studies indicate that the subunits interact through several distinct interfaces, including the PAS and coiled-coil regions as well as the catalytic domains. In the current study we have expressed the catalytic domains, attempting to generate active heterodimers for crystallization. As reported by others, the isolated catalytic domains of the α1 and ß1 subunits of human sGC associate predominantly as homodimers of either α or ß subunits, [51]. Neither homodimer has significant cyclase activity; however, when mixing the subunits there is an exchange with the heterodimeric form, as evidenced by the appearance of guanylate cyclase activity (ref. [51] and table 2). The homodimer of catalytic domains of the ß subunits crystallizes readily, but we have been unable to generate crystals of the heterodimer, possibly because of the co-existence of heterodimers and β homodimers. Based on the crystal structure of the ß homodimer and modeling of an α/ß dimer structure, we attempted to stabilize the heterodimeric form by engineering an S-S bond between the α and ß subunits. This involved introducing a cysteine residue in position 476^ß^, which was predicted to lie close to Cys595^α^ in the α subunit (in the following text, residues in the α and β subunits are indicated by a superscript following the number). Cys541^ß^, which is in the position homologous to Cys595^α^, was changed to Ser to prevent S-S bonds forming in the ß homodimer. As a result of these changes, we have been able to purify the α/β heterodimer as the predominant form after mixing the separately-expressed subunits. However, as reported earlier for the full-length protein [52], [53], the C541^β^S mutation is detrimental for activity.

**Table 2:** Guanylate cyclase activity of full-length and recombinant proteins.

Protein	Metal cofactor	Activity (this report) nmol/mg·min	Activity (published) nmol/mg·min	Comments
**Full-length heterodimer**	Mg	4300	10,000 [62]	
**Mixed catalytic domains**	Mn	25–300 depending on protein concentration	10–30 [51]	No activity for α2 or β2 homodimers
**Heterodimer of catalytic domains: sGCα+sGCβ^G476C/C541S^**	Mn	22		No activity with Mg
**Full-length heterodimer α/β(C541S)**	(Mg?)		40-fold less than WT [63]	

We describe here two crystal structures. The 1.63 Å crystal structure of the sGCβcat homodimer consists of residues 413–610 with some disorder at 440–441, where the structure was not modeled due to lack of electron density. The 2.08 Å structure of the α1/ß1 heterodimer consists of *GUCY1A3* residues 470–661 and *GUCY1B3* residues 413–608, with two engineered mutations in the ß1 subunit, G476^ß^ C and C541^ß^ S.

**Figure 2:**
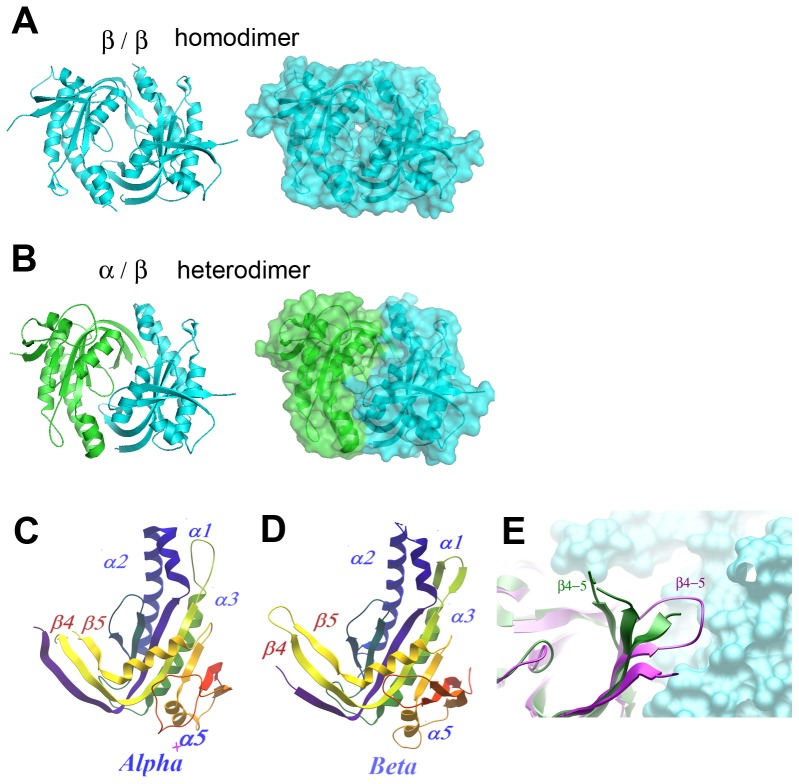
Overview of crystal structures. A. Homodimer of sGCβ catalytic domain (PDB ID: 2WZ1). B. Heterodimer of sGCα (green) and sGCβ (cyan) catalytic domains (PDB ID: 3UVJ). C. Architecture of the sGCα catalytic domain. D. Architecture of the sGCβ catalytic domain. E. The β4–5 loop in AC-C1 (purple) and sGCα (green); the surface of the β/C2 subunit is shown in cyan.

The homodimer and the heterodimer have a very similar fold and subunit interactions. The subunits come together to form a head-to-tail, wreath-like dimer, characteristic of adenylate cyclase [18], [39], [54] and the other sGC [10], [35] structures (figs. 2A, B). The structures of the α (fig. 2C) and ß subunits (fig. 2D) are highly similar. The monomers consist of a seven-stranded, ß-sheet core, surrounded by five alpha-helices, which were denoted as per the convention of adenylate and guanylate cyclase structures [10], [18], [35]. The main structural elements are the same or very similar in length and organization, with only subtle but possibly very important differences (see below). In particular, the α subunit features an extended β4–β5 hairpin which, in the heterodimer structure, points away from the β subunit; this is in contrast with other cyclase structures, in which the hairpin is involved in interdomain contacts (Fig. 2E). PISA [55] analysis of the interfaces of both homo- and heteo-dimer crystal structures report complexation significance score (CSS), a measure of predicted physiological relevance, as 1.000, indicating that the interface should be physiologically relevant. The buried surface area of the homodimer is 1454 Å^2^ while that of the heterodimer is 1283 Å^2^. Interestingly, the two cysteins (C476^β^ and C595^α^) that were expected to form a disulfide bridge are not linked in the crystal structure, although they are positioned in a distance that could allow an S-S bond. Since the introduction of C476^β^ resulted in the formation of stable heterodimers in solution, it is possible that an S-S bond may have broken during crystallization or data collection.

### Structural transitions

It has been proposed that class III nucleotidyl cyclases undergo a substrate-induced structural transition into a catalytically active “closed” state [18], [56], [57]. Upon substrate binding the α subunit (or its equivalent C1 domain of adenylate cyclase) rotates and translocates around the centre of the heterodimer, causing helix α1of the α subunit to move towards helix α4 of the ß subunit (C2 domain of AC), ‘closing’ the structure and bringing the catalytic residues into the effective positions for nucleotide cyclization.

The structure of the substrate-free sGC heterodimer presented here appears to be in an inactive open conformation, as is the chlamydomonas sGC structure [35]. Comparison of an ‘open’ apo-AC structure [54] with a ‘closed’ ligand -bound (2′3′ dideoxyATP) ACcat [18] structure shows a 7° rotation of the C1 monomer. Comparing the open conformation of the chlamydomonas structure with the closed conformation of the cyanobacteria homodimeric sGC shows a similar level of domain rotation 7–8° [10], [35]. However, aligning the subunits of the human sGC with the active adenylate cyclase structure requires a significantly larger rotation of approximately 26°, indicating that the conformational changes are more pronounced for human sGC.

**Figure 3:**
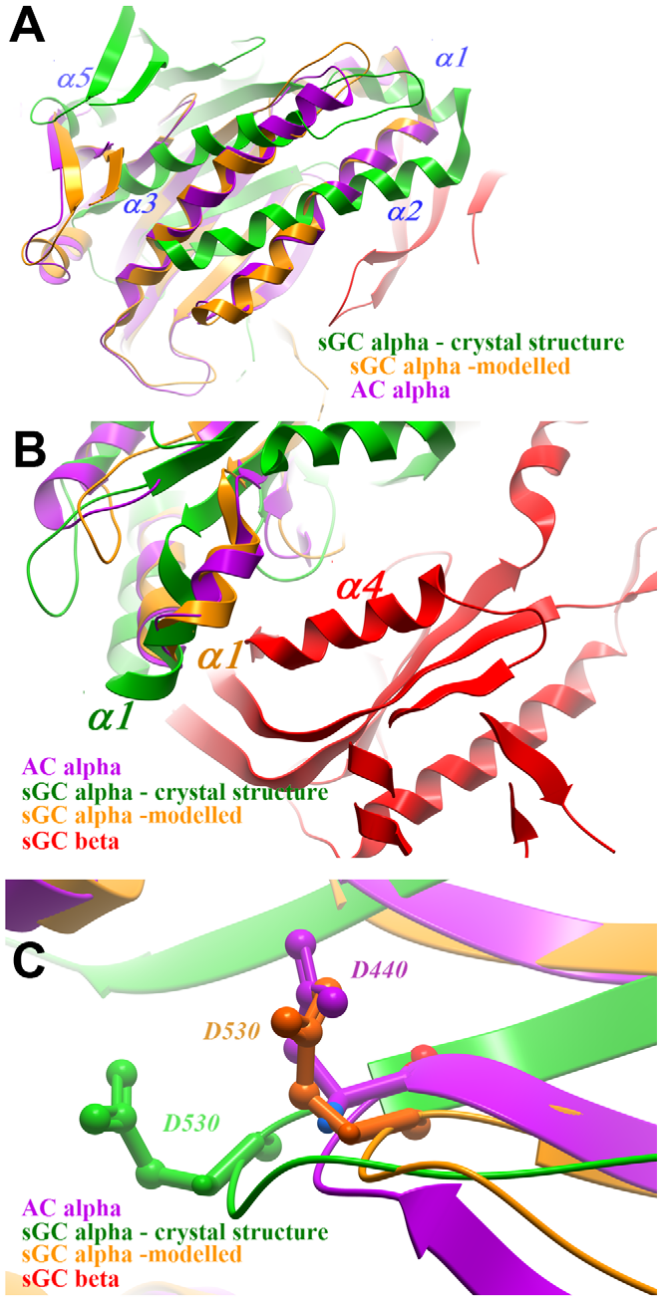
Structural transitions in sGC activation. A. sGCα in the crystal structure (green) compared with the same subunit (orange) modelled by alignment with the C1 domain of adelylate cyclase (purple). The rigid-body transition involves a 26° rotation, seen in the relative angles of the corresponding α helices. B. Detail: the change in position of the α1 helix (sGCα), bringing it closer to helix α4 (sGCβ). C. Detail: shift in position of the β6–7 loop, which brings a catalyitic residue D530 closer to the position of the corresponding residue in AC(D440).

Figure 3 shows details of the presumed transition between the crystal structure and the modeled active conformation. The 26° rigid-body rotation of the α subunit (Fig. 3A) involves movements of up to 10 Å in the positions of individual residues. For example, helix α1 (sGCα) shifts 7 Å in the direction of its N-terminal, towards helix α4 of sGCß (Fig. 3B). Another specific shift is a movement of the ß2–ß3 loop of sGCα, which contains the catalytic residue D530; This side chain retracts towards the position of the corresponding residue (D440) of AC (Fig. 3C). The consequences of the structural rearrangements on the location of active-site residues are discussed below.

### The α and ß subunits make distinct amino-acid contributions to the catalytic core of the heterodimer

**Figure 4:**
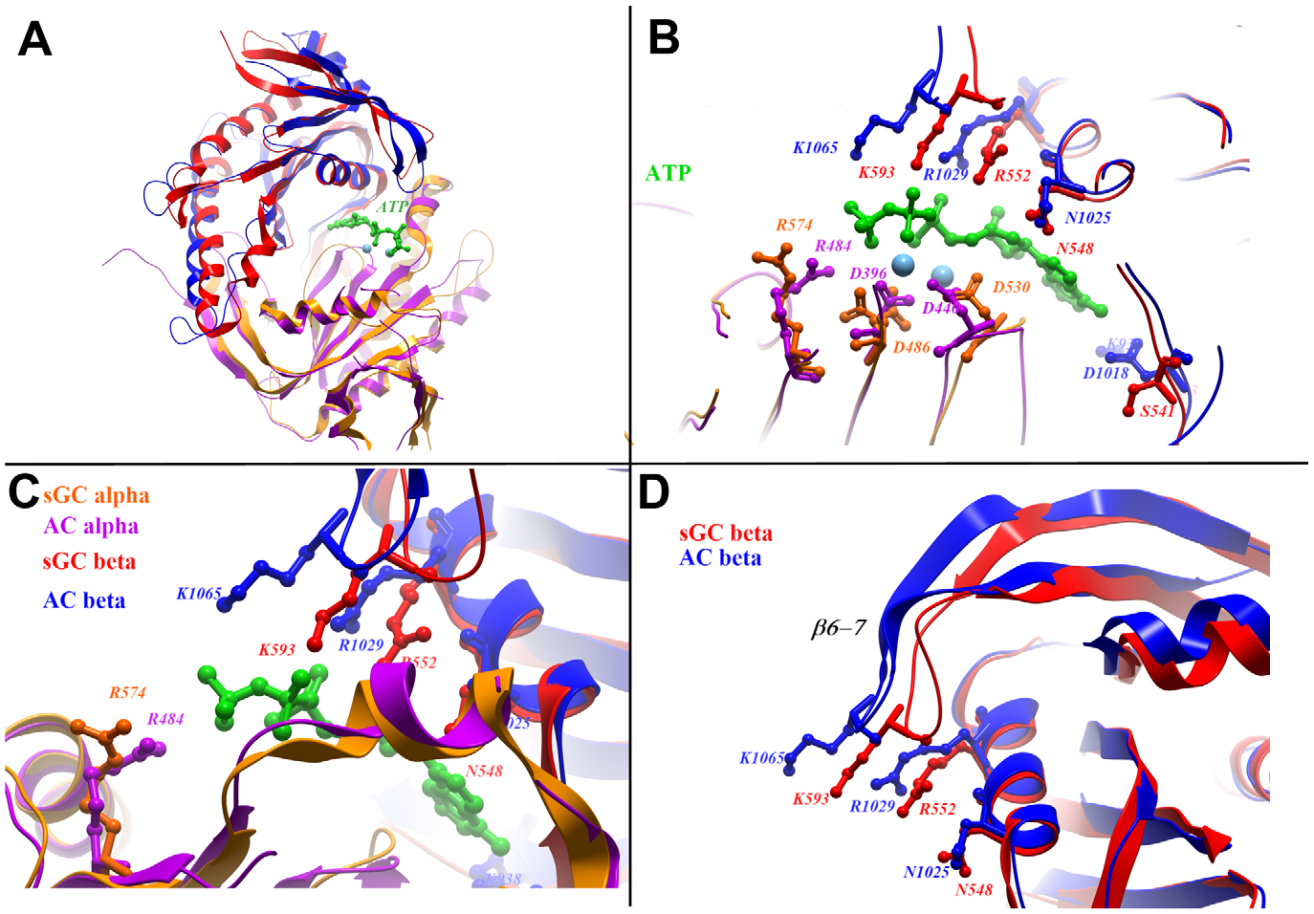
Active site residues on sGC in the modelled active conformation. The sGCα and sGCβ were separately aligned with AC domains C1 and C2, respectively. A. Overall view (the colour scheme is described in panel C). C. Active site residues surrounding an ATP analogue in the AC structure. C, D. Detailed views.

As discussed above, the crystal structure of the α/β heterodimer is in a conformation that is likely to be inactive. To analyze the locations of the active-site residues in a subunit arrangement that is more similar to the active conformation of adenylate cyclases, the sGC α and β subunits were separately superposed onto the C1 and C2 domains of an adenylate cyclase structure in the ATP-bound conformation (PDB ID:1CJK) [18]. The overall structural alignment of AC and the rearranged sCG is shown in Fig. 4A, with an ATP analogue positioned as in the AC structure.

Fig. 1 presents a summary of conserved residues that form part of the active site; the residue positions are generically marked 1–8, with the actual residues in sGC and AC listed in the 1B and 1C (a full sequence aligment is shown in Fig S1). Six of the eight residues are highly conserved between various AC and sGC variants.

Fig. 4B presents the location of the conserved residues in the aligned structures of AC and the rearranged sGC, surrounding the ATP and metal ions from the AC structure. Aspartates in positions 1 and 3 (residues D486 and D530 of the sGCα subunit) are essential for metal coordination. The electron density at D486 is best interpreted as a superposition of different rotamers of the side chain in the crystal structure (Fig. S2), indicating an inherent flexibility at this site, which probably becomes ordered upon metal-binding.

Residues in positions 4, 7 and 8 (R574 of sGCα, R552 and K593 of sGCß, respectively) are expected to stabilize the γ-phosphate of the substrate. R552^sGCß^ co-localizes with the equivalent residue in the AC structure (R1029), while the side chain of R574 (α) is pointing away from the proposed binding site. A loop (β5–β6) of the beta subunit is shifted inwards in sGCβ compared to the AC-C2 domain; consequently, residue K593^sGCβ^ does not overlap with the corresponding K1065 of AC. This loop may change position upon binding of nucleotide or upon physiological activation of the cyclase; alternatively, the altered position may enable a different interactions with the triphosphate moiety. Residue N548 of sGCß overlaps with the equivalent residue N1025 in AC (position 6), which binds the ribose moiety.

Positions 2 and 5 differ between AC and sGC proteins, and underlie the preference for adenosine and guanosine nucleotides, respectively. E473^sGCß^ and C541^sGCß^ are required for binding to the guanine of GTP, presumably with the cysteine forming a hydrogen bond to the O6 of guanine with the glutamate accepting hydrogen bonds from the N1 and N2 of guanine [58]. In AC, the corresponding residues are K938 (C2) and D1018 (C2), respectively, and it has been shown that mutating the GC residues to these AC residues switches the selectivity of the nucleotide to ATP, effectively turning a GC in to an AC [52], [59]. The C541^β^S mutation, which we introduced to obtain stable α/β heterodimers, has been shown to severely diminish GC activity [53]. This is despite the conservative nature of the substitution and the fact that serine occupies this position in the catalytic domain of the particulate guanylate cyclase in the protozoa Paramecium [60]. It has previously been suggested that the issue with this substitution is a steric one, rather than one involving hydrogen bonding to the ligand [35]. Comparison of the β subunit structures in the homodimer (which does not contain the substitution) and heterodimer shows an almost identical position of both the backbone and side-chain orientation of C541β and S541β.

**Figure 5:**
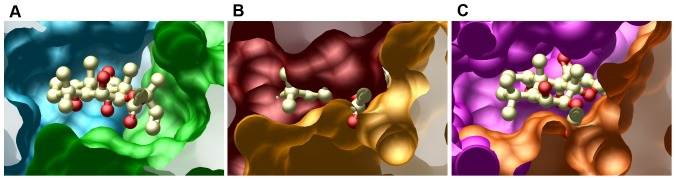
A possible allosteric site. A. Forskolin in its binding pocket in adenylate cyclase. B. The same region in sGC in the crystal structure: the cavity is collapsed, with no space for small-molecule binding. C. sGC in the modelled active conformation: a cavity opens up; although forskolin does not fit, other small molecules may occupy the site.

As discussed above, the dimeric nature of cyclases creates a cavity, which is pseudo-symmetrically related to the active site. In adenylate cyclase, this site can be partially occupied by the activator forskolin, which stabilizes an active conformation of the enzyme. The forskolin-binding cavity of AC is shown in Fig. 5A. In the crystal structure of the sGC heterodimer, the analogous cavity is collapsed (Fig. 5B). However, in the modeled active conformation of sGC, where each subunit is separately aligned with the corresponding domain of AC, a cavity is created (Fig. 5C). Although it does not fit into the cavity, the model illustrates that is a clear possibility of controlling the conformation (and hence the activity) of sGC through allosteric binding of small molecules.

## Discussion

This work presents the first structures of a metazoan, heteromeric guanylate cyclase. These structures can best be interpreted by comparing with known structures of adenylate cyclases and of guanylate cyclases of lower organisms.

The full-length human sGC occurs as a heterodimer of α and ß subunits. Truncated proteins containing only the catalytic domains associate predominantly as inactive α_2_ and ß_2_ homodimers. We show here that the structures of the β_2_ homodimer and the αβ heterodimer are remarkably similar, utilizing the same interaction surface. In fact, the buried surface area in the β_2_ dimer is larger than that of the αβ dimer. This raises the question of the determinants of heterodimerisation. In our experiments, the formation of stable heterodimers was driven by engineered mutations in the beta subunit; in the full-length native protein, heterodimerisation is likely to be driven by other domains of the protein.

A region immediately upstream of the catalytic domain has been shown to play a role in dimer formation, and was predicted to form an α-helical coiled-coil (this region, denoted as CC, includes residues 406–469 of sGCα and 346–409 of sGCβ). A crystal structure of the CC region of sGCβ shows an association of 4 α-helices, representing two anti-parallel coiled-coils [61]. The authors argue that this arrangement is unlikely to exist within a full-length dimer, as the distance between the C-termini of the helicase (∼60 Å) is incompatible with the predicted distance between the N-termini of the catalytic domains (∼30 Å). Based on the sequence considerations, the authors suggest that the CC regions in an αβ dimer may preferentially be oriented in parallel, which would bring the C-termini of the helices to a distance which is compatible with the N-termini of the catalytic domains. The structures presented here are compatible with their proposal, showing that the distance between the N-termini of the catalytic domains is in fact <25 Å (Fig. S3). It may be that the preference for heterodimers of full-length sGC is driven by of the beta CC regions forming antiparallel homodimers, which prevents the catalytic subunits from associating tightly, while the parallel association of the α and β CC regions promotes the direct association of the catalytic domains. Even if true, this is unlikely to be the whole story, as the N-terminal PAS and HNOB domains have important roles in dimerization.

Comparison of the structures presented here of the ß homodimer and the aß heterodimer with structures of nucleotide-bound adenylate cyclase clearly demonstrate why the homodimers are inactive: the key residues involved in nucleotide and metal-binding are distributed between the two subunits, and the full binding site cannot be reconstituted in any of the homodimers. The heterodimer structure contains all the conserved residues that were shown to be part of the active sites of other adenylate and guanylate cyclases. The subunit association seen in the crystal structure is in a conformation that is likely to be inactive. We can model an active conformation by aligning separately the α and β subunits with the C1 and C2 domains of adenylate cyclase in an ATP-bound conformation; this involves a 26° rigid-body rotation and translation of the α subunit. Similar reorientation of the subunits have been seen when comparing “open” and “closed” conformations of other AC and GC enzymes, although the magnitude of rotation us usually only ∼7°. It is possible that the crystals structure and the modeled active structure of sGC represent a conformational switch that occurs in the full-length enzyme, possibly induced by substrate binding and/or by interaction with the regulatory haem-binding domain. It is also possible that the conformational space is restricted by the coiled-coil structures upstream of the catalytic domains, which could create a constraint on the distance between the N-termini of the catalytic domains.

Three interesting observations emerge from this study and from structural work on other nucleotide cyclases. First, there is a remarkable conservation of a rather rigid structural framework that places the highly conserved active-site residues in overlapping locations within each subunit. However, there is considerable scope for changes in the relative orientation of the subunits, which provides a conformational switch that can be utilized for allosteric regulation.

Secondly, we have shown that the subunits of sGC can associate promiscuously as (inactive) homodimers or as (active) heterodimers using the same interaction surface. The preference for heterodimeric association must be involve other domains of the protein, but may also be promoted by binding of substrate or of other ligands. Further mutational and functional studies of the full-length protein may help to map regulatory interactions onto the structure, and help to understand the mechanisms involved.

Thirdly, the conformational change to the active state of sGC may be linked to the opening of a cavity in the region opposite the active site. It is possible that such a cavity may provide a binding site for small-molecule regulators of sGC activity. The crystal structures provide a framework for rational design of compounds targeting both the GTP-binding site and this potential allosteric site.

## Supporting Information

Figure S1 **Sequence alignment of the catalytic domains of selected guanylate and adenylate cyclases.** Sequences are presented with the letter of each scientific name of the organism. The molecules are denoted as being simply Guanylate Cyclase (GC) if the enzyme is active as a homodimer, and as GCA (alpha) or GCB (beta) if the enzyme is active as a heterodimer. Similarly, Adenylate Cyclase (AC) denotes an active homodimeric enzyme, while AC 1 and AC2 denote subdomains C1 and C2 of mammalian adenylate cyclases. Functional residues in the active site are highlighted as follows: metal binding (red); base-specifying (cyan); ribose binding (yellow) and triphosphate-stabilising (green). Sequences and GenBank accession IDs are as follows. *H.sapiens* GC alpha 1 (GCA) (AAB94794), *D.melanogaster* GCA (Q07093), *Canis familiaris* adenylate cyclase subunit 1 (AC1) (1CJU_A), *Homo sapiens* guanylate cyclase beta 1 (GCB) (2WZ1_A), *Drosophila melanogaster* GCB (Q9VA09), *R.norvegicus* AC2 (1CJU_B), *Chlamydomonas reinhardtii* GCB (3ET6_A), *D.melanogaster* GC (Q0KI72), *Spirulina platensis* AC (1WC1_C).
(PDF)

Figure S2 **Alternate conformations of sGCa^D486^.** The electron density can be best modelled as partial occupancy of different rotamers.
(TIF)

Figure S3 **Constraints on the multi-domain structure of GUCY.** Models of the PAS domain dimer (according to PDB ID:2P04)), the anti-parallel coiled-coil (PDB ID:3HLS) and the catalytic domains (this work, PDB ID:3UVJ). Dotted lines indicate the distances between the C-termini of the PAS domains (18 Å), the N-termini of the CC domains (82.3 Å), the C-termini of the CC domains (59.3 Å) and the N-termini of the catalytic domains (25.3 Å). Given these distances, it is unlikely that the CC helicase in the heterodimer are in an antiparallel orientation [61]. Furthermore, the rigid coiled-coil domain is likely to affect the relative orientation, and hence the activity state, of the catalytic domains.
(JPG)

## References

[1] Poulos TL (2006) Soluble guanylate cyclase. Curr Opin Struct Biol 16: 736–743. 17015012 10.1016/j.sbi.2006.09.006

[2] Mergia E, Russwurm M, Zoidl G, Koesling D (2003) Major occurrence of the new alpha2beta1 isoform of NO-sensitive guanylyl cyclase in brain. Cell Signal 15: 189–195. 12464390 10.1016/s0898-6568(02)00078-5

[3] Russwurm M, Koesling D (2002) Isoforms of NO-sensitive guanylyl cyclase. Mol Cell Biochem 230: 159–164. 11952091

[4] Mergia E, Koesling D, Friebe A (2009) Genetic mouse models of the NO receptor ‘soluble’ guanylyl cyclases. Handb Exp Pharmacol 33–46. 19089324 10.1007/978-3-540-68964-5_3

[5] Friebe A, Mergia E, Dangel O, Lange A, Koesling D (2007) Fatal gastrointestinal obstruction and hypertension in mice lacking nitric oxide-sensitive guanylyl cyclase. Proc Natl Acad Sci U S A 104: 7699–7704. 17452643 10.1073/pnas.0609778104 PMC1863512

[6] Schmidtko A, Gao W, Konig P, Heine S, Motterlini R, et al (2008) cGMP produced by NO-sensitive guanylyl cyclase essentially contributes to inflammatory and neuropathic pain by using targets different from cGMP-dependent protein kinase I. J Neurosci. 28: 8568–8576. 10.1523/JNEUROSCI.2128-08.2008 PMC6671070 18716216

[7] Saino M, Maruyama T, Sekiya T, Kayama T, Murakami Y (2004) Inhibition of angiogenesis in human glioma cell lines by antisense RNA from the soluble guanylate cyclase genes, GUCY1A3 and GUCY1B3. Oncol Rep 12: 47–52. 15201957

[8] Morton DB, Langlais KK, Stewart JA, Vermehren A (2005) Comparison of the properties of the five soluble guanylyl cyclase subunits in Drosophila melanogaster. J Insect Sci 5: 12. 16341244 10.1093/jis/5.1.12 PMC1307573

[9] Fitzpatrick DA, O'Halloran DM, Burnell AM (2006) Multiple lineage specific expansions within the guanylyl cyclase gene family. BMC Evol Biol 6: 26. 16549024 10.1186/1471-2148-6-26 PMC1435932

[10] Winger JA, Derbyshire ER, Lamers MH, Marletta MA, Kuriyan J (2008) The crystal structure of the catalytic domain of a eukaryotic guanylate cyclase. BMC Struct Biol 8: 42. 18842118 10.1186/1472-6807-8-42 PMC2576301

[11] Morton DB, Anderson EJ (2003) MsGC-beta3 forms active homodimers and inactive heterodimers with NO-sensitive soluble guanylyl cyclase subunits. J Exp Biol 206: 937–947. 12582136 10.1242/jeb.00160

[12] Roy B, Garthwaite J (2006) Nitric oxide activation of guanylyl cyclase in cells revisited. Proc Natl Acad Sci U S A 103: 12185–12190. 16882726 10.1073/pnas.0602544103 PMC1567716

[13] Ignarro LJ (1991) Heme-dependent activation of guanylate cyclase by nitric oxide: a novel signal transduction mechanism. Blood Vessels 28: 67–73. 1672101 10.1159/000158845

[14] Ma X, Sayed N, Baskaran P, Beuve A, van den Akker F (2008) PAS-mediated dimerization of soluble guanylyl cyclase revealed by signal transduction histidine kinase domain crystal structure. J Biol Chem 283: 1167–1178. 18006497 10.1074/jbc.M706218200 PMC3010369

[15] Yazawa S, Tsuchiya H, Hori H, Makino R (2006) Functional characterization of two nucleotide-binding sites in soluble guanylate cyclase. J Biol Chem 281: 21763–21770. 16754683 10.1074/jbc.M508983200

[16] Chang FJ, Lemme S, Sun Q, Sunahara RK, Beuve A (2005) Nitric oxide-dependent allosteric inhibitory role of a second nucleotide binding site in soluble guanylyl cyclase. J Biol Chem 280: 11513–11519. 15649897 10.1074/jbc.M412203200

[17] Lamothe M, Chang FJ, Balashova N, Shirokov R, Beuve A (2004) Functional characterization of nitric oxide and YC-1 activation of soluble guanylyl cyclase: structural implication for the YC-1 binding site? Biochemistry 43: 3039–3048. 15023055 10.1021/bi0360051

[18] Tesmer JJ, Sunahara RK, Gilman AG, Sprang SR (1997) Crystal structure of the catalytic domains of adenylyl cyclase in a complex with Gsalpha.GTPgammaS. Science 278: 1907–1916. 9417641 10.1126/science.278.5345.1907

[19] Whisnant RE, Gilman AG, Dessauer CW (1996) Interaction of the two cytosolic domains of mammalian adenylyl cyclase. Proc Natl Acad Sci U S A 93: 6621–6625. 8692867 10.1073/pnas.93.13.6621 PMC39075

[20] Yan SZ, Hahn D, Huang ZH, Tang WJ (1996) Two cytoplasmic domains of mammalian adenylyl cyclase form a Gs alpha- and forskolin-activated enzyme in vitro. J Biol Chem 271: 10941–10945. 8631912 10.1074/jbc.271.18.10941

[21] Surmeli NB, Marletta MA (2012) Insight into the Rescue of Oxidized Soluble Guanylate Cyclase by the Activator Cinaciguat. Chembiochem 10.1002/cbic.201100809 PMC3477619 22474005

[22] Martin F, Baskaran P, Ma X, Dunten PW, Schaefer M, et al (2010) Structure of cinaciguat (BAY 58-2667) bound to Nostoc H-NOX domain reveals insights into heme-mimetic activation of the soluble guanylyl cyclase. J Biol Chem 285: 22651–22657. 20463019 10.1074/jbc.M110.111559 PMC2903410

[23] Mitrovic V, Jovanovic A, Lehinant S (2011) Soluble guanylate cyclase modulators in heart failure. Curr Heart Fail Rep 8: 38–44. 21207207 10.1007/s11897-010-0045-1

[24] Garthwaite J, Southam E, Boulton CL, Nielsen EB, Schmidt K, et al (1995) Potent and selective inhibition of nitric oxide-sensitive guanylyl cyclase by 1H-[1,2,4]oxadiazolo[4,3-a]quinoxalin-1-one. Mol Pharmacol 48: 184–188. 7544433

[25] Morbidelli L, Pyriochou A, Filippi S, Vasileiadis I, Roussos C, et al (2010) The soluble guanylyl cyclase inhibitor NS-2028 reduces vascular endothelial growth factor-induced angiogenesis and permeability. Am J Physiol Regul Integr Comp Physiol 298: R824–832. 20032260 10.1152/ajpregu.00222.2009

[26] Olesen SP, Drejer J, Axelsson O, Moldt P, Bang L, et al (1998) Characterization of NS 2028 as a specific inhibitor of soluble guanylyl cyclase. Br J Pharmacol 123: 299–309. 9489619 10.1038/sj.bjp.0701603 PMC1565161

[27] Pyriochou A, Beis D, Koika V, Potytarchou C, Papadimitriou E, et al (2006) Soluble guanylyl cyclase activation promotes angiogenesis. J Pharmacol Exp Ther 319: 663–671. 16940434 10.1124/jpet.106.108878

[28] Zhu H, Li JT, Zheng F, Martin E, Kots AY, et al (2011) Restoring soluble guanylyl cyclase expression and function blocks the aggressive course of glioma. Mol Pharmacol 80: 1076–1084. 21908708 10.1124/mol.111.073585 PMC3228529

[29] Kajiya K, Huggenberger R, Drinnenberg I, Ma B, Detmar M (2008) Nitric oxide mediates lymphatic vessel activation via soluble guanylate cyclase alpha1beta1-impact on inflammation. FASEB J 22: 530–537. 17855621 10.1096/fj.07-8873com

[30] Majumder S, Rajaram M, Muley A, Reddy HS, Tamilarasan KP, et al (2009) Thalidomide attenuates nitric oxide-driven angiogenesis by interacting with soluble guanylyl cyclase. Br J Pharmacol 158: 1720–1734. 19912234 10.1111/j.1476-5381.2009.00446.x PMC2801213

[31] Amini A, Moghaddam SM, Morris DL, Pourgholami MH (2012) The critical role of vascular endothelial growth factor in tumor angiogenesis. Curr Cancer Drug Targets 12: 23–43. 22111836 10.2174/156800912798888956

[32] Cook KM, Figg WD (2010) Angiogenesis inhibitors: current strategies and future prospects. CA Cancer J Clin 60: 222–243. 20554717 10.3322/caac.20075 PMC2919227

[33] Crawford TN, Alfaro DV, 3rd, Kerrison JB, Jablon EP (2009) Diabetic retinopathy and angiogenesis. Curr Diabetes Rev 5: 8–13. 19199892 10.2174/157339909787314149

[34] Jeganathan VS (2011) Anti-angiogenesis drugs in diabetic retinopathy. Curr Pharm Biotechnol 12: 369–372. 20939797 10.2174/138920111794480525

[35] Rauch A, Leipelt M, Russwurm M, Steegborn C (2008) Crystal structure of the guanylyl cyclase Cya2. Proc Natl Acad Sci U S A 105: 15720–15725. 18840690 10.1073/pnas.0808473105 PMC2572937

[36] Tesmer JJ, Dessauer CW, Sunahara RK, Murray LD, Johnson RA, et al (2000) Molecular basis for P-site inhibition of adenylyl cyclase. Biochemistry 39: 14464–14471. 11087399 10.1021/bi0015562

[37] Mou TC, Masada N, Cooper DM, Sprang SR (2009) Structural basis for inhibition of mammalian adenylyl cyclase by calcium. Biochemistry 48: 3387–3397. 19243146 10.1021/bi802122k PMC2680196

[38] Mou TC, Gille A, Suryanarayana S, Richter M, Seifert R, et al (2006) Broad specificity of mammalian adenylyl cyclase for interaction with 2′,3′-substituted purine- and pyrimidine nucleotide inhibitors. Mol Pharmacol 70: 878–886. 16766715 10.1124/mol.106.026427

[39] Tesmer JJ, Sunahara RK, Johnson RA, Gosselin G, Gilman AG, et al (1999) Two-metal-Ion catalysis in adenylyl cyclase. Science 285: 756–760. 10427002 10.1126/science.285.5428.756

[40] Savitsky P, Bray J, Cooper CD, Marsden BD, Mahajan P, et al (2010) High-throughput production of human proteins for crystallization: the SGC experience. J Struct Biol 172: 3–13. 20541610 10.1016/j.jsb.2010.06.008 PMC2938586

[41] Battye TG, Kontogiannis L, Johnson O, Powell HR, Leslie AG (2011) iMOSFLM: a new graphical interface for diffraction-image processing with MOSFLM. Acta Crystallogr D Biol Crystallogr 67: 271–281. 21460445 10.1107/S0907444910048675 PMC3069742

[42] Evans P (2006) Scaling and assessment of data quality. Acta Crystallogr D Biol Crystallogr 62: 72–82. 16369096 10.1107/S0907444905036693

[43] Emsley P, Cowtan K (2004) Coot: model-building tools for molecular graphics. Acta Crystallogr D Biol Crystallogr 60: 2126–2132. 15572765 10.1107/S0907444904019158

[44] Murshudov GN, Vagin AA, Dodson EJ (1997) Refinement of macromolecular structures by the maximum-likelihood method. Acta Crystallogr D Biol Crystallogr 53: 240–255. 15299926 10.1107/S0907444996012255

[45] Adams PD, Afonine PV, Bunkoczi G, Chen VB, Davis IW, et al (2010) PHENIX: a comprehensive Python-based system for macromolecular structure solution. Acta Crystallogr D Biol Crystallogr 66: 213–221. 20124702 10.1107/S0907444909052925 PMC2815670

[46] Painter J, Merritt EA (2006) Optimal description of a protein structure in terms of multiple groups undergoing TLS motion. Acta Crystallogr D Biol Crystallogr 62: 439–450. 16552146 10.1107/S0907444906005270

[47] Long F, Vagin AA, Young P, Murshudov GN (2008) BALBES: a molecular-replacement pipeline. Acta Crystallogr D Biol Crystallogr 64: 125–132. 18094476 10.1107/S0907444907050172 PMC2394813

[48] Bricogne G. BE, Brandl M., Flensburg C., Keller P., Paciorek W.,, Roversi P SA, Smart O.S., Vonrhein C., Womack T.O. (2011) BUSTER version 2.10.0. Cambridge, United Kingdom: Global Phasing Ltd.

[49] Chen VB, Arendall WB 3rd, Headd JJ, Keedy DA, Immormino RM, et al (2010) MolProbity: all-atom structure validation for macromolecular crystallography. Acta Crystallogr D Biol Crystallogr 66: 12–21. 20057044 10.1107/S0907444909042073 PMC2803126

[50] Poornam GP, Matsumoto A, Ishida H, Hayward S (2009) A method for the analysis of domain movements in large biomolecular complexes. Proteins 76: 201–212. 19137621 10.1002/prot.22339

[51] Winger JA, Marletta MA (2005) Expression and characterization of the catalytic domains of soluble guanylate cyclase: interaction with the heme domain. Biochemistry 44: 4083–4090. 15751985 10.1021/bi047601d

[52] Sunahara RK, Beuve A, Tesmer JJ, Sprang SR, Garbers DL, et al (1998) Exchange of substrate and inhibitor specificities between adenylyl and guanylyl cyclases. J Biol Chem 273: 16332–16338. 9632695 10.1074/jbc.273.26.16332

[53] Friebe A, Russwurm M, Mergia E, Koesling D (1999) A point-mutated guanylyl cyclase with features of the YC-1-stimulated enzyme: implications for the YC-1 binding site? Biochemistry 38: 15253–15257. 10563809 10.1021/bi9908944

[54] Zhang G, Liu Y, Ruoho AE, Hurley JH (1997) Structure of the adenylyl cyclase catalytic core. Nature 386: 247–253. 9069282 10.1038/386247a0

[55] Krissinel E, Henrick K (2007) Inference of macromolecular assemblies from crystalline state. J Mol Biol 372: 774–797. 17681537 10.1016/j.jmb.2007.05.022

[56] Sinha SC, Wetterer M, Sprang SR, Schultz JE, Linder JU (2005) Origin of asymmetry in adenylyl cyclases: structures of Mycobacterium tuberculosis Rv1900c. EMBO J 24: 663–673. 15678099 10.1038/sj.emboj.7600573 PMC549627

[57] Sinha SC, Sprang SR (2006) Structures, mechanism, regulation and evolution of class III nucleotidyl cyclases. Rev Physiol Biochem Pharmacol 157: 105–140. 17236651 10.1007/112_0603

[58] Hurley JH (1998) The adenylyl and guanylyl cyclase superfamily. Curr Opin Struct Biol 8: 770–777. 9914257 10.1016/s0959-440x(98)80097-3

[59] Tucker CL, Hurley JH, Miller TR, Hurley JB (1998) Two amino acid substitutions convert a guanylyl cyclase, RetGC-1, into an adenylyl cyclase. Proc Natl Acad Sci U S A 95: 5993–5997. 9600905 10.1073/pnas.95.11.5993 PMC27573

[60] Linder JU, Hoffmann T, Kurz U, Schultz JE (2000) A guanylyl cyclase from Paramecium with 22 transmembrane spans. Expression of the catalytic domains and formation of chimeras with the catalytic domains of mammalian adenylyl cyclases. J Biol Chem 275: 11235–11240. 10753932 10.1074/jbc.275.15.11235

[61] Ma X, Beuve A, van den Akker F (2010) Crystal structure of the signaling helix coiled-coil domain of the beta1 subunit of the soluble guanylyl cyclase. BMC Struct Biol 10: 2. 20105301 10.1186/1472-6807-10-2 PMC2828450

[62] Humbert P, Niroomand F, Fischer G, Mayer B, Koesling D, et al (1990) Purification of soluble guanylyl cyclase from bovine lung by a new immunoaffinity chromatographic method. Eur J Biochem 190: 273–278. 1973095 10.1111/j.1432-1033.1990.tb15572.x

[63] Friebe A, Wedel B, Harteneck C, Foerster J, Schultz G, et al (1997) Functions of conserved cysteines of soluble guanylyl cyclase. Biochemistry 36: 1194–1198. 9063867 10.1021/bi962047w

