# Supplementary material for: Crystal Structures of the Catalytic Domain of Human Soluble Guanylate Cyclase
Source: PLoS One. 2013 Mar 7;8(3):e57644. doi: 10.1371/journal.pone.0057644 (PMC3591389; doi:10.1371/journal.pone.0057644)
Supplement: Figure S1 — Sequence alignment of the catalytic domains of selected guanylate and adenylate cyclases. Sequences are presented with the letter of each scientific name of the organism. The molecules are denoted as being simply Guanylate Cyclase (GC) if the enzyme is active as a homodimer, and as GCA (alpha) or GCB (beta) if the enzyme is active as a heterodimer. Similarly, Adenylate Cyclase (AC) denotes an active homodimeric enzyme, while AC 1 and AC2 denote subdomains C1 and C2 of mammalian adenylate cyclases. Functional residues in the active site are highlighted as follows: metal binding (red); base-specifying (cyan); ribose binding (yellow) and triphosphate-stabilising (green). Sequences and GenBank accession IDs are as follows. H.sapiens GC alpha 1 (GCA) (AAB94794), D.melanogaster GCA (Q07093), Canis familiaris adenylate cyclase subunit 1 (AC1) (1CJU_A), Homo sapiens guanylate cyclase beta 1 (GCB) (2WZ1_A), Drosophila melanogaster GCB (Q9VA09), R.norvegicus AC2 (1CJU_B), Chlamydomonas reinhardtii GCB (3ET6_A), D.melanogaster GC (Q0KI72), Spirulina platensis AC (1WC1_C). (PDF) [file pone.0057644.s001.pdf]

```
                     480       490            500       510       520
Hs GCA   477   FSNVTMLFSDIVGFTAICSQCS----PLQVITMLNALYTRFDQQCGEL---DVYKVETIG
Dm GCA   462   YPDVTILFSDIVGFTSICSRAT----PFMVISMLEGLYKDFDEFCDFF---DVYKVETIG
Cf AC1   387   HDNVSILFADIEGFTSLASQCT----AQELVMTLNELFARFDKLAAEN---HCLRIKILG
Hs GCB   417   YDNVTILFSGIVGFNAFCSKHASGEGAMKIVNLLNDLYTRFDTLTDSRKNPFVYKVETVG
Dm GCB   415   YDSVTLMFSGIVGFGQYCAANTDPDGAMKIVKMLNELYTVFDALTDSKRNLNVYKVETVG
Rn AC2   882   YDCVCVMFASIPDFKEFYTESDVNKEGLECLRLLNEIIADFDDLLSKPKFSGVEKIKTIG
Cr GC    473   HPEATVLFSDIVGFTEIASRSS----PLEVCSLLDELYQRFDAAIEEYP--QLYKVETIG
Dm GC    417   FDSVSILFSDIVTFTEICSRIT----PMEVVSMLNAMYSIFDKLTERN---SVYKVETIG
Sp AC     1008  PRLITILFSDIVGFTRMSNALQ----SQGVAELLNEYLGEMTRAVFEN---QGTVDKFVG
                         1                                              2

               530                  540       550       560            570
Hs GCA   530   DAYCVAGGLH-----------KESDTHAVQIALMALKMMELSDEVMS-PHG-----EPIK
Dm GCA   515   DAYCVASGLH-----------RASIYDAHKVAWMALKMIDACSKHIT-HDG-----EQIK
Cf AC1   440   DCYYCVSGLP-----------EARADHAHCCVEMGMDMIEAISLVREMTG------VNVN
Hs GCB   477   DKYMTVSGL------------PEPCIHHARSICHLALDMMEIAGQVQVDGE-------SVQ
Dm GCB   587   DKYMAVSGL------------PDHCEDHAKCMARVALDMMDMAKNVKMGSN-------PVQ
Rn AC2   942   STYMAATGLSAIPSQEHAQEPERQYMHIGTMVEFAYALVGKLDAINKHSFN------DFK
Cr GC    527   DAYMVVCNVT-----------VPCDDHADVLLEFALRMHEEASRVAS-SLG-----EPVR
Dm GC    470   DAYMVVAGAP-----------DKDANHAERVCDMALDMVDAITDLKDPSTG-----QHLR
Sp AC     1061  DAIMALYGAPEEMSP-----SEQVRRAIATARQMLVALEKLNQGWQERGLVGRNEVPPVR
               3

                      580         590       600       610       620       630
Hs GCA   573   MRIGLHSGSVFAGVVG-VKMPRYCLFGNNVTLANKFESCSVPRKINVSPTTYRLLKDCPG
Dm GCA   558   MRIGLHTGTVLAGVVG-RKMPRYCLFGHSVTIANKFESGSEALKINVSPTTKDWLTKHEG
Cf AC1   483   MRVGIHSGRVHCGVLG-LRKWQFDVWSNDVTLANHMEAGGKAGRIHITKATLSYLNGDY-
Hs GCB   515   ITIGIHTGEVVTGVIG-QRMPRYCLFGNTVNLTSRTETTGEKGKINVSEYTYRCLMSPEN
Dm GCB   629   ITIGIHSGEVVTGVIG-NRVPRYCLFGNTVNLTSRTETTGVPGRINVSEETYRLLCMAIN
Rn AC2   996   LRVGINHGPVIAGVIG-AQKPQYDIWGNTVNVASRMDSTGVLDKIQVTEETSLILQTLG-
Cr GC    570   IRVGMHSGPVVAGVVG-RKMPRFCLFGDTVNTASRMESHGEAGQIHISEACYCCLRSKE-
Dm GC    514   IRVGVHSGAVVAGIVG-LKMPRYCLFGDTVNTASRMESTSIAMKVHISESTKVLIGPN--
Sp AC     1116  FRCGIHQGMAVVGLFGSQERSDFTAIGPSVNIAARLQEATAPNSIMVSAMVAQYVPDEE-
                4                     5      6   7

                   630       640         650       660       670       680
Hs GCA   632   F--VFTPRSREELPPNFPSEIPGICHFLDAYQQGTNSKPCFQKKDVEDGNANFLGKASGI
Dm GCA   617   F--EFELQPRD--PSFLPKEFP--------NPGGTET--CY-----------FLESFR--
Cf AC1   541   ---EVEPGCGGERNAYLKEHSIETFLILRCTQKRKEEKAMIAK-----------------
Hs GCB   578   SDPQFHLEHRGPVSMKGKKEPMQVWFLSRKNT----GTEETKQDDDAEN-----LYFQ--
Dm GCB   688   QDDSFHLEYRGPVIMKGKPTPMDCWFLTRATSSILGGTSSTGGSGGGGNGSLDSPLLQPA
Rn AC2   1054  ----YTCTCRGIINVKGKGD-LKTYFVNTEMSR---------------------------
Cr GC    628   ---RFEIRERGNITVKGKGTMR-------TYLLSPLERT---------------------
Dm GC    571   ----YKIIERGEIDVKGKGTMG------TYWLEEREN-----------------------
Sp AC     1175  ------IIKREFLELKGIDEPVMTCVINPNMLNQ--------------------------
                              8
```
