# Supplementary figures and images for: Crystal Structures of the Catalytic Domain of Human Soluble Guanylate Cyclase
Source: PLoS One. 2013 Mar 7;8(3):e57644. doi: 10.1371/journal.pone.0057644 (PMC3591389; doi:10.1371/journal.pone.0057644)

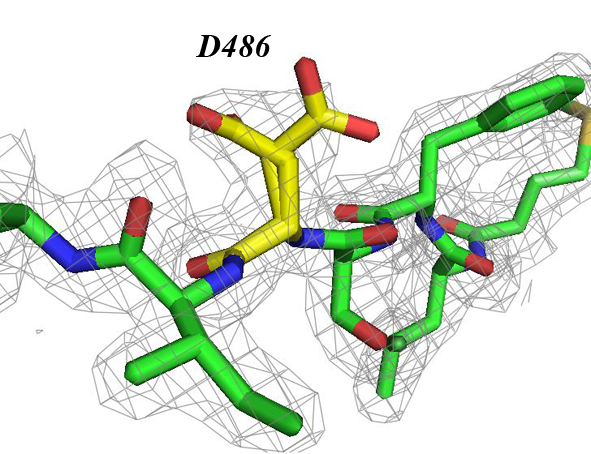

Supplement: Figure S2 — Alternate conformations of sGCaD486. The electron density can be best modelled as partial occupancy of different rotamers. (TIF) [file pone.0057644.s002.tif]

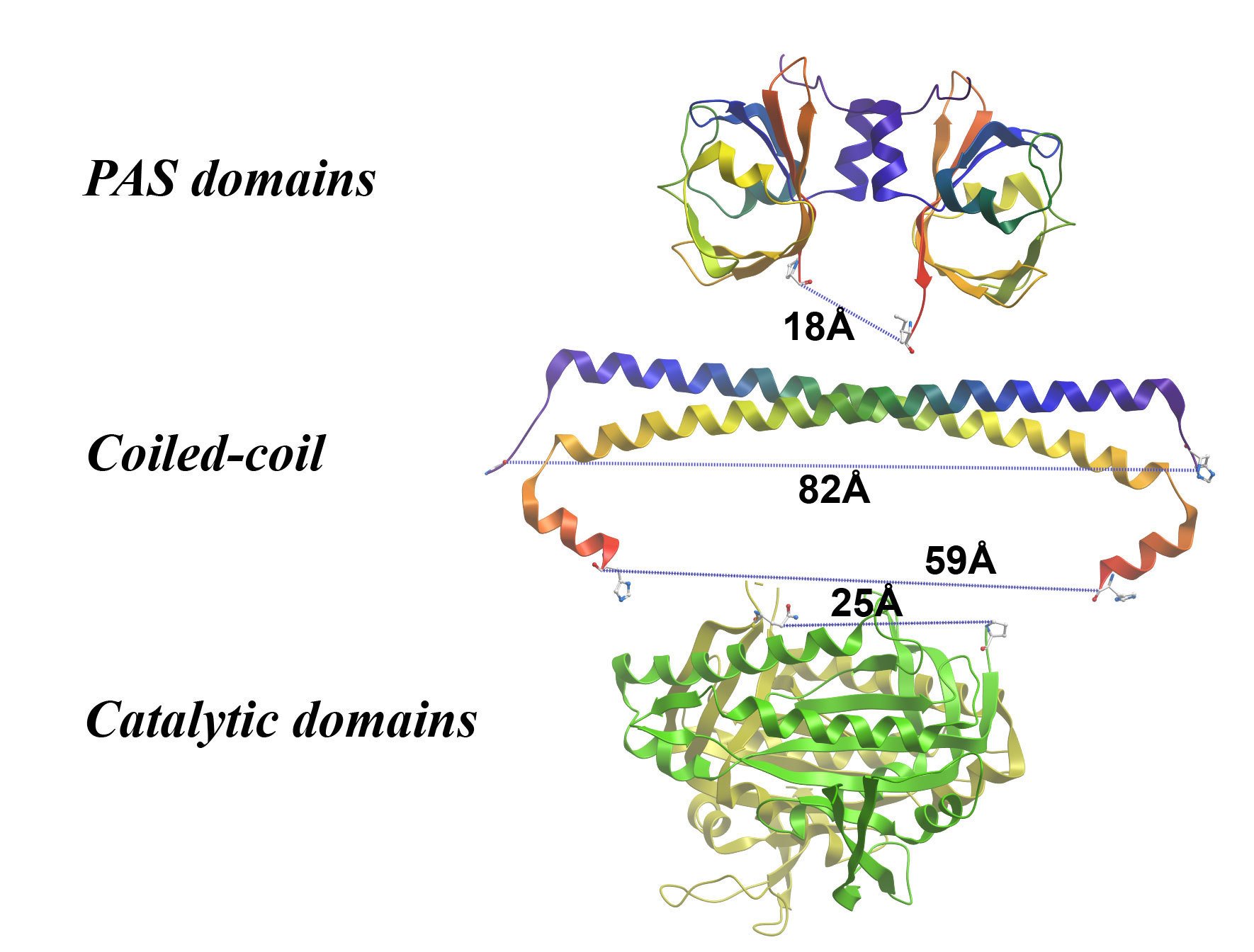

Supplement: Figure S3 — Constraints on the multi-domain structure of GUCY. Models of the PAS domain dimer (according to PDB ID:2P04)), the anti-parallel coiled-coil (PDB ID:3HLS) and the catalytic domains (this work, PDB ID:3UVJ). Dotted lines indicate the distances between the C-termini of the PAS domains (18 Å), the N-termini of the CC domains (82.3 Å), the C-termini of the CC domains (59.3 Å) and the N-termini of the catalytic domains (25.3 Å). Given these distances, it is unlikely that the CC helicase in the heterodimer are in an antiparallel orientation [61]. Furthermore, the rigid coiled-coil domain is likely to affect the relative orientation, and hence the activity state, of the catalytic domains. (JPG) [file pone.0057644.s003.jpg]
